# ID 46 - Desenvolvimento de Ferramenta de Caracterização Técnica de Profissionais de Avaliação de Tecnologias em Saúde no Âmbito das Secretarias Estaduais de Saúde do Brasil

**DOI:** 10.5327/2237-9622.2025.v34s1.46

**Published:** 2025-11-25

**Authors:** Marina Petrasi Guahnon, Bruna Marmett, Ana Paula Blankenheim, Bárbara Cristiane da Silva, Rodrigo Pereira de Almeida, Roseana Boek Carvalho, Muriel Primon de Barros, Gilson Pires Dorneles, Suena Medeiros Parahiba, Maicon Falavigna

**Keywords:** Secretarias Estaduais de Saúde, saúde baseada em evidência, tomada de decisão

## Abstract

**Introdução:**

No contexto nacional, a Avaliação de Tecnologias em Saúde (ATS) é um tema que vem sendo explorado nos últimos anos. A implementação efetiva dos processos de ATS requer a atuação de profissionais com habilidades e conhecimentos específicos. Atualmente, há ausência de dados de caracterização técnica de profissionais que atuam em ATS dentro de Secretarias Estaduais de Saúde (SES) e não há uma forma padronizada de mapear esta informação. Portanto, desenvolvemos uma ferramenta para o mapeamento da caracterização profissional de servidores estaduais que atuam na área de ATS.

**Método:**

O desenvolvimento da ferramenta para mapeamento da caracterização técnica de profissional que atuam em ATS nas SES consistiu em duas etapas. Na primeira etapa, foi elaborado um formulário de caracterização técnica dos profissionais (direcionado aos que atuam em ATS) com base no formulário da Rebrats e oficina para discutir os pontos com a Rebrats. Na segunda etapa, foram incluídas as perguntas do Instrumento para Avaliação da Saúde Baseada em Evidências (I-SABE), o qual considera ações dos profissionais em relação à prática de saúde baseada em evidências e discussão com especialista para a melhor aplicabilidade do questionário. A ferramenta foi elaborada no Google Forms e os dados serão compilados em banco de dados no programa Microsoft Excel. A análise de dados ocorrerá com a utilização do software R em conjunto ao R Studio. O projeto de pesquisa foi aprovado pelo Comitê de Ética em Pesquisa (CEP) da Associação Hospitalar Moinhos de Vento sob número de Parecer 6.963.930.

**Resultados:**

A ferramenta elaborada contempla sete seções. O Termo de Consentimento Livre e Esclarecido eletrônico foi inserido no início do formulário, condicionando o seguimento do preenchimento. As primeiras seis seções incluem questões referentes aos seguintes dados: informações pessoais; dados funcionais; formação acadêmica; produção científica; conhecimentos gerais; dedicação à ATS. Posteriormente, seguem as questões referentes ao I-SABE, incluindo cinco domínios: autoeficácia; comportamento; atitude; resultados/benefícios e conhecimento/habilidade.

**Conclusão:**

A ferramenta desenvolvida pode ser utilizada para o mapeamento de dados referentes à formação, à capacitação e à experiência no desenvolvimento de produtos de ATS. Sendo de suma importância para o melhor entendimento do perfil de profissionais atuando em ATS no contexto estadual.

